# Current status and future perspectives of HLA-edited induced pluripotent stem cells

**DOI:** 10.1186/s41232-020-00132-9

**Published:** 2020-10-01

**Authors:** Keiko Koga, Bo Wang, Shin Kaneko

**Affiliations:** 1 Takeda-CiRA Joint Program (T-CiRA), 2-26-1, Muraoka-Higashi, Fujisawa, Kanagawa 251-8555 Japan; 2 T-CiRA discovery, Takeda Pharmaceutical Company, 2-26-1, Muraoka-Higashi, Fujisawa, Kanagawa 251-8555 Japan; 3 Shin Kaneko Laboratory, Department of Cell Growth and Differentiation, Center for iPS cell research (CiRA), Kyoto University, 53 Shogoin Kawahara-cho, Sakyo-ku, Kyoto, 606-8507 Japan

## Abstract

In 2007, Human-induced pluripotent stem cells (iPSCs) were generated by transducing four genes (Oct3/4, Sox2, Klf4, c-Myc). Because iPSCs can differentiate into any types of cells in the body and have fewer ethical issues compared to embryonic stem (ES) cells, application of iPSCs for regenerative medicine has been actively examined. In fact, iPSCs have already been used for clinical applications, but at present, only autologous iPSC-derived grafts or HLA homozygous iPSC-derived grafts are being transplanted into patients following HLA matching. HLA is an important molecule that enables the immune system differentiates between self and non-self-components; thus, HLA mismatch is a major hurdle in the transplantation of iPSCs. To deliver inexpensive off-the-shelf iPSC-derived regenerative medicine products to more patients, it is necessary to generate universal iPSCs that can be transplanted regardless of the HLA haplotypes. The current strategy to generate universal iPSCs has two broad aims: deleting HLA expression and avoiding attacks from NK cells, which are caused by HLA deletion. Deletion of B2M and CIITA genes using the CRISPR/Cas9 system has been reported to suppress the expression of HLA class I and class II, respectively. Transduction of NK inhibitory ligands, such as HLA-E and CD47, has been used to avoid NK cell attacks. Most recently, the HLA-C retaining method has been used to generate semi-universal iPSCs. Twelve haplotypes of HLA-C retaining iPSCs can cover 95% of the global population. In future, studying which types of universal iPSCs are most effective for engraftment in various physiological conditions is necessary.

## Background

Pluripotent ES cells are used in regenerative medicine as source cells [1]. However, they were deemed unethical as their preparation involves the destruction of embryos. The introduction of human iPSCs prepared from fibroblasts and blood cells in a relatively non-invasive manner in 2007 [2] has led to an increase in their feasibility for clinical application. In 2014, the world’s first surgery was performed to transplant a sheet of retinal pigment epithelial cells derived from the iPSCs of a patient with age-related macular degeneration. The 2-year follow-up revealed that the transplanted sheet had remained intact, and the patient’s visual acuity did not worsen [3]. However, due to the high costs and large amounts of time required to generate patient-specific iPSCs, it is necessary to prepare off-the-shelf iPSC-derived grafts. A major hurdle in the clinical application of off-the-shelf iPSCs is HLA (human leucocyte antigen) compatibility. HLA is a molecule that enables the immune system to distinguish between self and non-self-entities in the body [4], and HLA compatibility is positively correlated with graft survival rates after transplantation [5]. Therefore, to deliver off-the-shelf iPSC-derived grafts, it is necessary to generate universal iPSCs, such as transplantable iPSCs that are free from HLA compatibility issues. Recent discovery and application of the CRISPR/Cas9 system has made it possible to rapidly edit specific genes [6–9]. In this review, we summarize the reports on the HLA-edited iPSCs and discuss the potential challenges associated with this procedure.

**Fig. 1:**
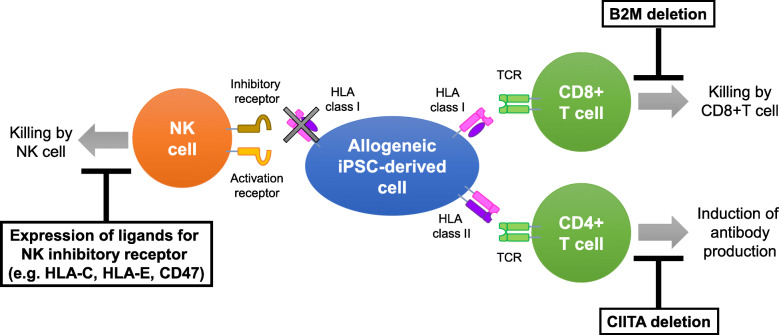
Schematic representation of the mechanisms for allogeneic iPSC-derived cells to evade immunological rejection. Allogeneic iPSC-derived cell are recognized and eliminated by allo-reactive T cells through HLA class I and class II molecules. Deletion of HLA class I and class II are achieved by B2M and CIITA gene deletion, respectively. On the other hand, loss of HLA class I molecules, which function as ligands for NK inhibitory receptors, causes attack by NK cells. To avoid killing by NK cells, expressions of NK inhibitory ligands, such as HLA-C, HLA-E and CD47 have been reported

## Main text

### Human leucocyte antigen (HLA)

Human leucocyte antigen (HLA) is a gene group that was originally identified as one of the determinants of the blood types of white blood cells and was subsequently identified as human major histocompatibility complex (MHC) [4]. The HLA genes are located within a 3-Mbp region of the short arm of chromosome 6 [10]. HLA genes are divided into two classes: HLA-class I and HLA-class II. Class I comprises three major genes, HLA-A, B, and C, and three minor genes, HLA-E, F, and G. HLA-class II consists of three genes, HLA-DR, DQ, and DP. The HLA proteins encoded by the HLA genes are expressed on the surface of the cell membrane and function as heterodimers made of two proteins. Each gene of HLA class I encodes the alpha chain and forms heterodimers with the the β2 microglobulin protein encoded by the B2M gene. HLA class II genes encode both the alpha and beta chains [4]. The HLA genes are the most polymorphic genes among all human genes; there are tens of thousands of combinations of these genes, which account for differences seen across various individuals [11].

The functions of HLA can be divided into two broad categories. HLA class I and class II form complexes with antigen peptides, interact with TCRs of CD8+ T cells and CD4+ T cells, and present antigens. This HLA-peptide-TCR interaction initiates the antigen-specific adaptive immune response. They also act as a ligand for T cells and NK cells for the recognition of self/non-self-components in the body. T cells recognize cells that express non-self HLA, whereas NK cells recognize cells that do not express self HLA. Based on these mechanisms, non-self-cells are eliminated from the body. The mechanisms by which T cells and NK cells eliminate non-self-cells are described in the following paragraph.

T cells that strongly react with self HLA are eliminated by negative selection during their development in thymus, while a part of T cells that undergo positive selection by self HLA and appear in the periphery recognize non-self HLA [12]. Such T cells are called allo-reactive T cells; they are activated through interactions with cells expressing non-self HLA. Activated allo-reactive CD4 + T cells induce the production of allo-specific antibodies by B cells, and allo-reactive CD8+ T cells directly attack and kill interacting cells [13, 14].

On the other hand, NK cells eliminate cells that do not express self HLA. NK cells express receptors on their cell surface; their responses to target cells are regulated by the extent of the stimulation of NK cell-specific activation or inhibitory ligands. Since HLA class I molecules function as inhibitory ligands for NK cells, cells that do not display HLA class I molecules are attacked and killed by NK cells [15]. Aptotosis and perforin-induced cell lysis are some of the common mechanisms used by both NK cells and CD8 + T cells to kill target cells [16].

There are several NK inhibitory receptors, and they can be further divided into subcategories such as the receptors for HLA molecules and the common inhibitory receptors with T cells. The former consists of the KIR (killer immunoglobulin-like receptor) family, CD94/NKG2A heterodimer, and ILT2 (LILRB1/CD85j), and the latter consists of TIGIT, CD96, KLRG1, PD1, and Tim3. HLA-C molecules are classified into two groups: C1 and C2. Several inhibitory KIRs, such as KIR2DL2/L3 and KIR2DL1, are receptors for C1 and C2, respectively. Other inhibitory KIRs, such as KIR3DL1 and KIR3DL2, also function as inhibitory receptors bind to specific HLA-A or HLA-B. Minor HLA class I molecules, HLA-E, HLA-F, and HLA-G, function as ligands for CD94/NKG2A, KIR2DL2, and KIR3DL4, respectively. ILT2 interacts with various HLA class I molecules including HLA-A, B, C, E, and G [17, 18]. The expression patterns of NK inhibitory receptors are heterogenous [19], i.e., each NK inhibitory receptor is not expressed on all NK cells. Therefore, it is not easy to suppress NK cell activation in its entirety.

### Significance of HLA-edited iPSCs for allogenic transplantation

The results from the analysis of clinical data in transplantation medicine highlight the importance of HLA matching in organ transplantation. The importance and contribution of each HLA in kidney transplantation have been analyzed in several studies. Of the six major HLAs (A, B, C, DP, DQ, DR), HLA-DR is the highest contributor to HLA compatibility [20], followed by HLA-A and HLA-B [9]. It has also been reported that matching of HLA-DQ and HLA-C is positively correlated with engraftment rates [21, 22]. On the other hand, there is also a report that the contribution of HLA-DP towards HLA compatibility is low [23]. The meta-analysis of heart transplantation has revealed that matching of HLA-DR significantly increases the graft survival rates [24]. These findings indicate that higher HLA compatibility also leads to better engraftment rate and therapeutic results for transplantation with allogeneic iPSC-derived cells, along with lower dosage of immunosuppressants. However, the safest method is to prepare autologous iPSCs, allow their differentiation into desired cells, and transplant these cells into the patient. High costs and longer preparation times are the two major drawbacks of this method.

An alternative method uses iPSCs derived from HLA homozygotes. It is considered that 40% of the Japanese population can be covered by the stocks of HLA homozygous iPSCs, as they display relatively fewer HLA polymorphisms [25]. In clinical studies, allogeneic iPSC-derived corneal epithelial cells of HLA homozygotes have been transplanted into HLA-matched patients (follow-up pending). The iPSCs from HLA homozygotes have lower manufacturing costs, rapid distribution, and undergo thorough quality control assessments. However, this method is only suitable for the Japanese population. Additionally, studies using monkey MHC homozygous iPSCs have revealed that the grafts derived from MHC homozygous iPSCs have different engraftment rates depending on the cell type, in the absence of immunosuppressants. Although dopamine neurons function by engrafting iPSC-derived cells homozygous without immunosuppressants [26], cardiomyocytes require immunosuppressants for their optimum functioning [27, 28]. These differences also contribute to immunogenicity of the organ/tissue. Therefore, there is a need to generate universal iPSCs (by modifying the HLA genes), which can be directly utilized for transplantation irrespective of HLA haplotypes.

### HLA-edited ES cells and iPSCs

Most strategies for HLA editing consist of two steps: complete suppression of HLA and expression of a molecule that can avoid NK cell attacks (Fig. 1).

In order to delete HLA expression in entirety, both HLA-I and HLA-II must be deleted. Since the region of the HLA-I/II gene is very large, it is difficult to delete it entirely using direct gene editing. The B2M gene, which is a common subunit forming a heterodimer, is deleted to effectively suppress the expression of HLA-class I [29]. Deletion of the CIITA gene, which is a transcription factor essential for HLA-II expression, is used for the suppression of HLA-II [30]. These two deletions lead to the formation of cells in which HLA is completely deleted. The allo-reactivity of T cells and NK cell-recognition has been analyzed in pluripotent and differentiated cells containing a deleted HLA-I, HLA-II, or both HLA-I/II. B2M-deficient ES cell-derived embryoid body cells show significant but not complete reduction of allo-reactivity by peripheral blood mononuclear cells (PBMCs) [29]. Lung alveolar epithelial cells derived from B2M-deficient ES cells and CD45+ hematopoietic cells can completely escape the allo-reaction by CD8+ T cells. However, NK cell activity is considerably enhanced by B2M deficiency [31]. Additionally, in vivo assays suggest that depletion of host NK cells might be required for the engraftment of B2M-deficient ES-derived cells [31, 32]. Both B2M and CIITA-deficient iPSC-derived cardiomyocytes and endothelial cells have also been generated and characterized [33, 34]. Cardiomyocytes derived from B2M/CIITA-deficient iPSCs can partially escape the allo-reaction by PBMCs. However, cardiomyocytes and endothelial cells derived from B2M/CIITA-deficient iPSCs enhance the activity of NK cells [33, 34]. B2M-deficient cells evade allo-reactions of CD8+ T cells but conversely enhance attack by NK cells, and CIITA deficiency reduces allo-reactions of CD4+ T cells.

In order to escape the attack from NK cells, forced expression of HLA-E and CD47, which function as NK inhibitory ligands, has been examined. ES cells, in which HLA-E fused with B2M were knock-in to B2M locus to delete endogenous B2M expression and other HLA-class I expression, enable differentiated CD45+ hematopoietic cells to overcome NK-mediated killing in vitro and in vivo [32]. As HLA-E acts as a ligand for the CD94/NKG2A heterodimer, which is expressed by the majority of NK cells, HLA-E expression might be sufficient to avoid an attack by NK cells. A study suggests that iPSC-derived cardiomyocytes and endothelial cells with overexpressed CD47 can avoid an attack by NK cells in vitro and show long-time survival in vivo [34]. CD47 is a highly expressed surface molecule in syncytiotrophoblast cells, which are present in the interface between maternal blood and fetal tissue; moreover, it is a ligand for SIPR alpha and also inhibits phagocytosis. However, NK cells do not express SIPR alpha, and receptors for CD47 on NK cells are yet to be studied. Another group deleted the major HLA class I (HLA-A/-B/-C) selectively by using the CRISPR/Cas9 system, deleted HLA class II molecules by editing the CIITA gene, and knocking in the immunoregulatory factors PD-L1, HLA-G, and CD47 in ES cells to control T cell-, NK cell-, and macrophage-mediated immune responses. In vitro and in vivo experiments from this study showed that T cell responses were suppressed, and NK cell killing and macrophage engulfment of the edited ES cell-derived endothelial cells and vascular smooth muscle cells was minimal [35]. Our recent study reports a strategy for deleting only HLA-A/B expression and retaining HLA-C expression using the CRISPR/Cas9 system [36]. We have demonstrated that iPSC-derived CD43+ blood cells with HLA-A/B deletion and unmodified HLA-C and HLA-E/F/G evade immune rejection from only HLA-C-matched NK cells and T cells, in in vitro and in vivo experiments. Moreover, deletion of CIITA in these cells suppresses CD4 + T cell activation. These results can be attributed to the fact that minor HLA class I molecules, i.e., HLA-E, F, and G, and HLA-C were intact in our study, unlike the B2M deletion. CIITA deletion and HLA-C matching suppress allo-reaction of CD4+ and CD8 + T cells, respectively, and both HLA-C and minor HLA class I molecules suppress NK activation through different NK inhibitory receptors. We also analyzed the frequencies of HLA-C alleles in Japanese, European, African, Asian, and Hispanic populations. We found that 12 frequent HLA-C alleles were sufficient to cover 95% of the global population, suggesting that 12 HLA-C alleles expressing iPSC lines could be used as semi-universal iPSCs.

### Future perspectives

At present, the biggest challenge for the establishment of strategies involving HLA class I/II deletion is the effective prevention of attacks from NK cells. This can be attained by either the suppression of NK-activating receptors or the activation of NK inhibitory receptors. There are multiple NK-activating receptors, and their expression levels are also different for different cell types. Therefore, it is difficult to suppress all NK-activating receptors. It is possible to suppress NK cells by activating NK inhibitory receptors; however, there are no inhibitory receptors that are expressed universally in all NK cells, and therefore, multiple inhibitory receptors need to be suppressed at the same time to achieve complete NK cell suppression. Furthermore, application of an agonist antibody against the inhibitory receptor could be useful as a method other than the enhanced expression of ligands specific for NK inhibitory receptors.

It will be necessary to compare and elucidate which universal iPSC candidates can avoid the attack from NK cells most efficiently under various physiological conditions in the body. Additionally, the current gene editing techniques utilize the CRISPR/Cas9 technology, but the safety of this technology also needs to be examined. In the future, if a technique capable of accurately and rapidly editing a large HLA gene region is developed, the whole HLA region of off-the-shelf iPSC-derived grafts could be edited to match the recipient’s HLA completely, right before transplantation. Another strategy to achieve the long-term engraftment of allogenic iPSC-derived grafts is to induce immunotolerance by immune cells. Regulatory immune cells such as hematopoietic stem cells (HSC), progenitor T cells (ProT), regulatory T cells (Tregs), and regulatory dendritic cells (regDCs) generated from the same iPSCs as the grafts may have the potential to achieve immunotolerance in vivo. Mixed chimerism has been used to confer immunotolerance to HSCs [37], and the potential for its incorporation in Tregs and regDCs is currently under investigation [38, 39].

## Conclusion

In order to rapidly and inexpensively provide iPSC-derived regenerative medicine products to patients, generation of universal iPSCs that are transplantable without the need for matching HLA types is necessary. With the recent development of the CRISPR/Cas9 system, it has become possible to easily edit genes, and the feasibility of HLA-edited iPSCs has been greatly enhanced. In addition, several methods to generate universal or semi-universal iPSCs that can avoid NK cell attacks due to HLA deficiency have already been reported. In the future, it is necessary to verify which universal iPSC candidates can engraft and function most efficiently under various physiological conditions in the body, because the optimal combination of universal iPSCs and differentiated cells may differ depending on the cell types. Establishment of universal iPSCs can rapidly advance the clinical application of regenerative medicine-based therapies using off-the-shelf iPSCs, and can contribute greatly to medical care.

## Data Availability

Not applicable

## Abbreviations

B2M: Beta 2 microglobulin
CIITA: Class II major histocompatibility complex transactivator
Cas9: CRISPR-associated protein 9
CRISPR: Clustered regularly interspaced short palindromic repeats
ES cell: Embryonic stem cell
HLA: Human leucocyte antigen
ILT: Immunoglobulin-like transcript
iPSC: Induced pluripotent stem cells
KIR: Killer immunoglobulin-like receptor
LILR: Leukocyte immunoglobulin-like receptor
MHC: Major histocompatibility complex
NK cell: Natural killer cell
SIPR alpha: Signal regulatory protein alpha
